# Multimodal ultrasound radiomics containing microflow images for the prediction of central lymph node metastasis in papillary thyroid carcinoma

**DOI:** 10.3389/fonc.2025.1604951

**Published:** 2025-07-16

**Authors:** Jiangyuan Ben, Qiying Yv, Pengfei Zhu, Junhao Ren, Pu Zhou, Guifang Chen, Ying He

**Affiliations:** ^1^ Cancer Research Center Nantong, Affiliated Tumor Hospital of Nantong University, and Medical School of Nantong University, Nantong, China; ^2^ Department of Ultrasound, Affiliated Tumor Hospital of Nantong University, Nantong, Jiangsu, China

**Keywords:** papillary thyroid cancer, multimodal ultrasound, microflow, elastography, lymph node metastasis, machine learning, radiomics

## Abstract

**Objectives:**

This study aimed to construct a model by applying radiomics and machine learning (ML) to multimodal ultrasound images (including grayscale, elastography and microflow images) along with clinical data to predict central lymph node metastasis (CLNM) in patients with papillary thyroid cancer (PTC).

**Methods:**

A cohort of 213 patients who underwent thyroidectomy accompanied by lymph node dissection (LND) and were pathologically diagnosed with PTC postoperatively was enrolled and randomized to the training cohort (n = 170) or testing cohort (n = 43). Radiomics features were extracted from multimodal images and subsequently screened via the least absolute shrinkage and selection operator (LASSO). The same methods were applied to screen clinical features. Nine ML algorithms were used to construct clinical models, radiomics models and fusion models. Model performance was assessed via receiver operating characteristic curves (ROC), decision curve analysis (DCA), and Delong test. Finally, the optimal model was interpreted and visualized via Shapley additive explanation (SHAP).

**Results:**

In each modality, 1561 features were extracted from the ultrasound images. Sixteen features were ultimately retained, including 6 grayscale features, 6 elastography features, and 4 microflow features. From the clinical features, including gender, age, traditional ultrasound signs and serological indicators, 2 relevant features were selected. Among the prediction models, the fusion model constructed by Multilayer Perceptron (MLP) algorithm showed the best diagnostic performance, outperforming the other models in both the training cohort (AUC = 0.886) and the testing cohort (AUC = 0.873).

**Conclusions:**

The fusion model based on clinical data and multimodal ultrasound radiomics has better predictive ability and net clinical benefit for CLNM in patients with PTC, confirms the diagnostic value of microflow images for CLNM, and can help to evaluate patients’ preoperative lymph node status and make the correct decision on the surgical procedure.

## 1 Introduction

The incidence of thyroid cancer is steadily increasing globally. According to the latest epidemiological data, there were more than 821,000 new cases of thyroid carcinoma worldwide in 2022, making it the seventh most common cancer in terms of overall incidence (1). Papillary thyroid carcinoma (PTC), which is the main pathological type of thyroid carcinoma, has the best overall prognosis (2). However, the incidence of cervical lymph node metastasis in PTC can be as high as 40–90% (3–6) Cervical lymph node metastasis is one of the major risk factors that increases the recurrence rate and decreases the survival rate of patients with PTC (7). Therefore, prophylactic central lymph node dissection (pCLND) is usually considered for PTC patients in China and some other Asian–Pacific countries to reduce the risk of reoperations.

Whether routine pCLND is an overtreatment has become one of the major controversies in PTC treatment (8). In clinical practice, pCLND often leads to complications such as laryngeal recurrent nerve injury, hypoparathyroidism and chyle leakage. According to the American Thyroid Association guidelines, thyroidectomy without pCLND can be considered for small, noninvasive, clinically node-negative PTCs and most follicular carcinomas (3). Hence, accurate preoperative assessment of cervical lymph node metastasis, especially central lymph node metastasis (CLNM), is crucial for surgical decision-making.

Currently, the preoperative assessment of cervical lymph nodes in patients with PTC relies on imaging techniques (mainly ultrasound) and fine needle aspiration (FNA). According to previous studies, due to the anatomical structure of the central neck, conventional ultrasound has a sensitivity of less than 55% in the preoperative diagnosis of CLNM, which is inferior to that of lateral lymph node metastasis (LLNM) (9, 10). Some metastatic lymph nodes lack typical malignant features, which may lead to false-negative diagnoses. FNA is an invasive test with limited accuracy that may be affected by the size of metastatic lesions and operators’ technical expertise, and therefore is not currently the preferred clinical method for evaluating CLNM (11, 12). Not all lymph nodes can be definitively diagnosed by FNA. Some patients yield non-diagnostic samples due to inadequate sampling, while others require further evaluation for indeterminate cytology results, including repeat FNA, molecular testing, or diagnostic excision. In addition, FNA may lead to certain adverse outcomes, such as hematoma formation and tumor cell seeding (13, 14). In recent years, multimodal ultrasound diagnosis, which combines ultrasound grayscale patterns, ultrasound Doppler patterns, ultrasound elastography patterns, and ultrasound microflow patterns, has been gradually promoted (15). Multimodal ultrasound offers a new perspective for disease diagnosis. Wu et al. and Li et al. demonstrated that elastography images and microflow images correlate with malignancy and LNM in PTC, but did not use a quantitative approach to analyze multimodal images (16, 17).

Radiomics allows quantitative features to be extracted from medical images for more precise analysis of lesions, which is in line with the trend toward precision medicine (18). The application of radiomics to multimodal ultrasound images has been reported to be effective in improving the diagnostic performance of ultrasound. Liu et al. applied radiomics to grayscale and Doppler images of endometrial cancer patients to create a multimodal ultrasound radiomics model for predicting LNM (19). However, there are no studies on the use of multimodal ultrasound to predict CLNM in thyroid cancer patients.

In our study, radiomics and machine learning were applied to multimodal ultrasound images, including grayscale images, elastography images and microflow images. Finally, multimodal ultrasound radiomics features were combined with clinical features to construct a machine learning (ML) model for the preoperative prediction of CLNM in PTC patients.

## 2 Materials and methods

### 2.1 Patients and data collection

**Figure 1:**
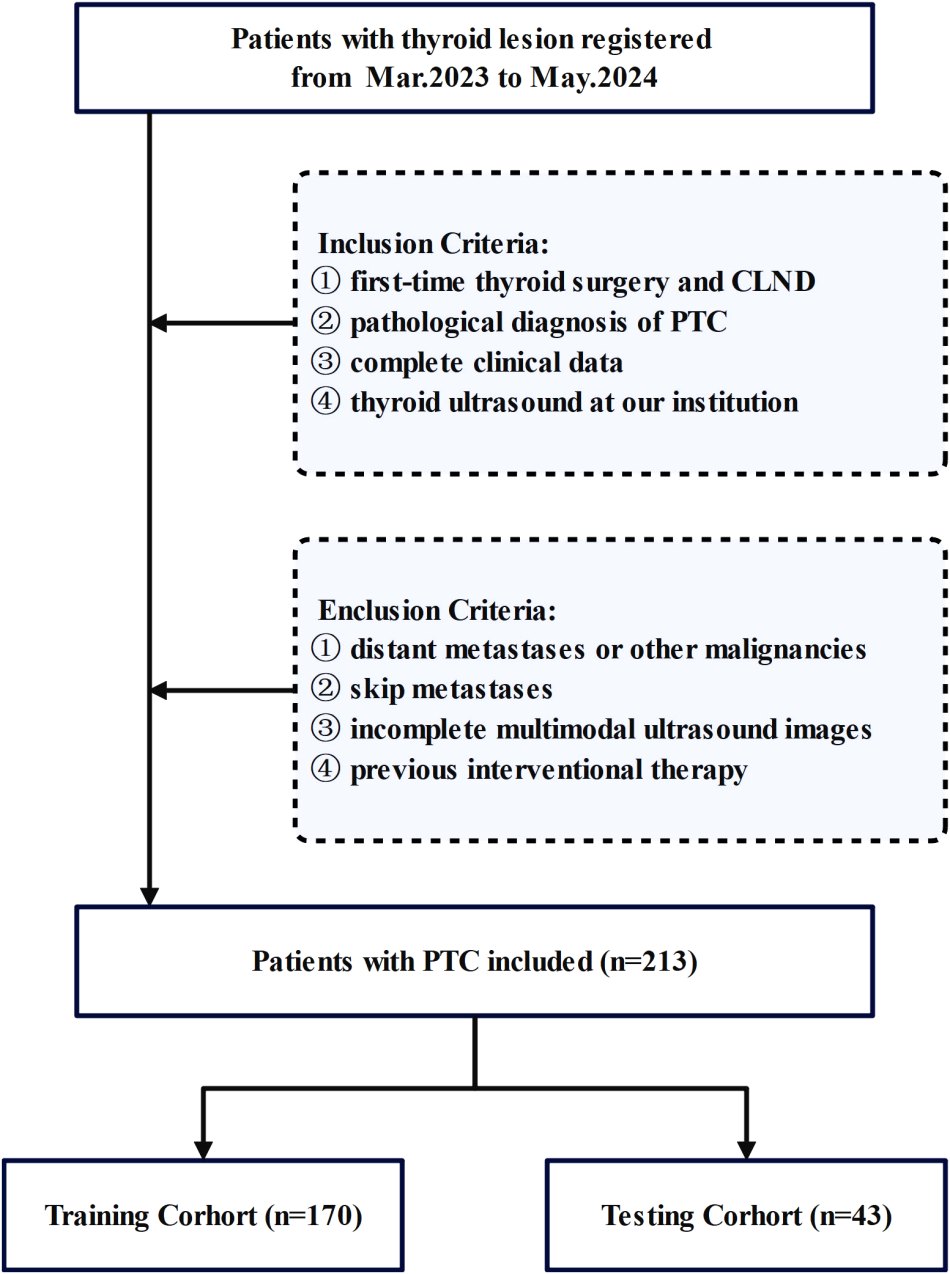
Flowchart of patient enrollment, including inclusion and exclusion criteria.

This study conducted a comprehensive review of medical records from February 2023 to June 2024 at the Affiliated Tumor Hospital of Nantong University. All patients with resectable papillary thyroid carcinoma (PTC) underwent pCLND according to current guidelines. The inclusion criteria were as follows: (1) first-time thyroid surgery and CLND, (2) postoperative pathological diagnosis of PTC, (3) complete clinical data and (4) thyroid ultrasound at our institution within 1 week before surgery. We excluded patients who (1) had distant metastases or other malignancies, (2) had skip metastases, (3) whose multimodal ultrasound images were incomplete, or (4) had undergone previous interventional therapy. The inclusion process is shown in **Figure 1**.

Finally, 213 patients with PTC who met specific criteria were included in the study. Basic clinical data, including age, lesion characteristics on ultrasound, and preoperative serological data, were collected. Patient data were randomly divided into a training set (n=170) and a testing set (n=43). The clinical baseline characteristics showed no significant differences between the training and validation sets, demonstrating their comparability. Cervical lymph node status was determined on the basis of postoperative pathology results.

The research adhered to the principles of the Declaration of Helsinki. All procedures were performed in accordance with established institutional protocols and regulatory standards. Given the retrospective nature of the investigation, the ethics committee granted a waiver for informed consent (approval identifier: 2024-097-07). Prior to the review, patients’ medical records were anonymized to remove any identifying information.

### 2.2 US image acquisition

Preoperative US images were acquired by a certified physician with more than 20 years of experience in thyroid ultrasound using a SAMSUNG (RS85) instrument equipped with a 3–12 MHz linear probe. The parameters of the ultrasound machine were fixed according to the routine requirements of thyroid ultrasound to obtain standard thyroid images. The specific parameters, including scanning depth sufficient to fully visualize the thyroid gland, resolution and frequency adequate to meet diagnostic requirements for radiologists, and other clinically relevant specifications, were determined based on the Chinese guidelines for the diagnosis and management of thyroid nodules and differentiated thyroid cancer (Second edition), along with previous relevant studies (20–22). For all PTC patients, multimodal US images displaying the lesion at its maximum diameter, including grayscale, elastography and microflow images, were acquired. All conventional ultrasound features were uniformly evaluated by a single radiologist with 20 years of experience in thyroid diagnosis, who was blinded to the pathological results, to eliminate inter-observer variability.

### 2.3 Ultrasound image segmentation and radiomics feature extraction

ITK-SNAP (version 3.8.0) was used to segment the ultrasound images manually. The region of interest (ROI) was independently segmented by radiologist 1, who has more than 10 years of experience in thyroid ultrasound. The radiologist outlined the entire tumor area along the lesion boundaries on grayscale, elastography and microflow images. After accurate segmentation of the ROIs, 1561 distinctive features were extracted from each modality using the PyRadiomics open-source tool (available at https://www.example.com/en/latest/index.html).

Two weeks later, 50 images of randomly selected cases were redrawn by radiologists 1 and 2, both of whom have more than 10 years of experience in thyroid ultrasound, and the features were then extracted again via the method described above. In order to evaluate inter- and intra-observer segmentation consistency, intragroup correlation coefficient (ICC) tests were performed within groups using radiomics features obtained at different times by radiologist 1 and between groups using radiomics features obtained by radiologists 1 and 2. Radiomics features with ICC values greater than 0.75 are considered to be stable features. We extracted the stable features from the images in the training cohort, and then these features were filtered by an independent t test after Z-score normalization. Subsequently, redundant features with thresholds above 0.9 in the Pearson correlation analysis were removed. The identified features are analyzed via the least absolute shrinkage and selection operator (LASSO), and the most important features are selected for CLNM prediction.

### 2.4 Establishment of the radiomics model

**Figure 2:**
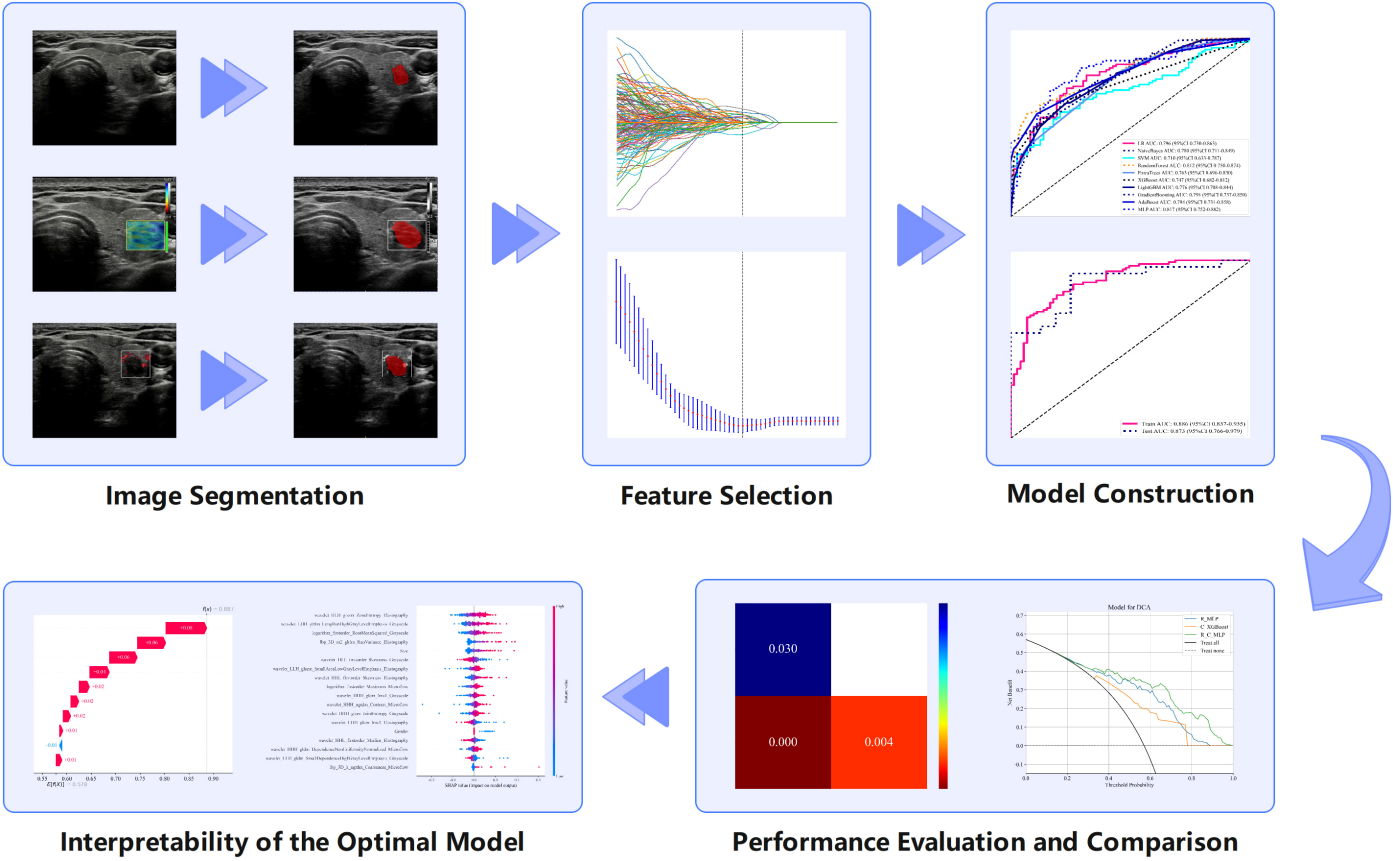
Radiomics analysis flowchart showing the various steps involved in radiomics research and examples of each step.

Nine ML algorithms—Logistic Regression (LR), Naive Bayes Classifier (NaiveBayes), Support Vector Machine (SVM), Random Forest Classifier (RandomForest), Extremely Randomized Trees (Extra Trees), Extreme Gradient Boosting (XGBoost), Light Gradient Boosting Machine (LightGBM), Adaptive Boosting (AdaBoost), and Multilayer Perceptron (MLP)—were utilized to analyze the radiomics features obtained through LASSO screening. For each modality, the model with the highest area under the curve (AUC) and its corresponding algorithm, which is deemed most suitable for that modality, are retained. The three algorithms identified from the unimodal ultrasound radiomics model selection were then applied to the construction of three multimodal ultrasound radiomics models, resulting in a total of nine models. Among these, the model exhibiting the highest AUC was selected as the definitive multimodal ultrasound radiomics model (R model), and the corresponding algorithm was then used to construct the clinical radiomics model (C-R model). The analysis process of radiomics is illustrated in **Figure 2**.

### 2.5 Establishment of the clinical model

Z-score normalization was also utilized on clinical features, and these features were subsequently filtered by independent t tests or Mann-Whitney U test. After removing redundant features with thresholds above 0.9 in the Pearson correlation analysis, the identified features were analyzed via LASSO, and the most important features were selected for CLNM prediction. Similar to the construction of the ultrasound radiomics model, nine algorithms were utilized to develop the clinical model (C model), and then the model with the highest AUC and its corresponding algorithm were selected.

### 2.6 Establishment of the clinical radiomics model

**Figure 3:**
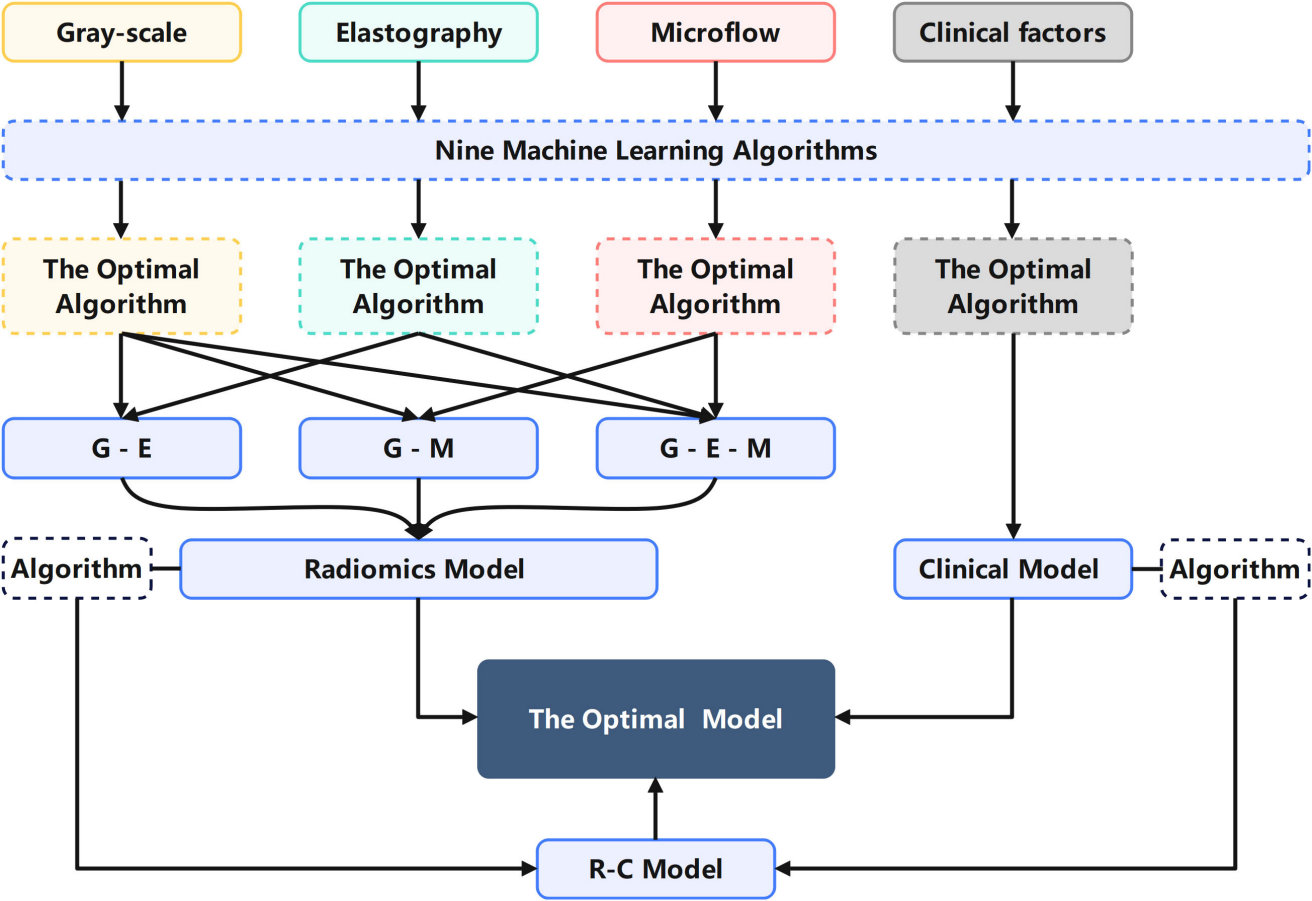
Mind map for the model construction in this study explaining the process of selecting algorithms and modalities.

All features, including multimodal ultrasound radiomics features and clinical features, underwent the same screening process as described above. The two algorithms most suitable for the R model and C model were applied to the construction of construct the C-R model, and the optimal model was selected. The mind map for the model construction in this study is depicted in **Figure 3**. We employed the DeLong test to compare the R-model, the C-model, and the C-R model. Decision curve analysis (DCA) was used to calculate and compare the net benefits at various threshold probabilities for both the training and validation cohorts, thereby assessing the clinical utility of the three models.

### 2.7 Interpretability of the optimal model

SHAP (Shapley additive explanation) was used to dissect the contribution of individual variables to the optimal model. SHAP addresses the inherent ‘black box’ nature of ML models by calculating the average marginal contribution to quantify each feature’s impact on the prediction (23, 24). By analyzing the importance of each feature and ranking them in descending order according to their respective SHAP values, the study identified key predictors, thereby improving the understanding of the complex relationship between CLNM and radiomics features.

### 2.8 Statistics

Statistical analyses were conducted using Python (version 3.7), R (version 4.2.0), and IBM SPSS Statistics 26.0 (IBM Corp, Armonk, NY, USA). Categorical variables and continuous variables are expressed as numbers and percentages and mean ± standard deviations, respectively. Independent t test were used to compare normally distributed continuous variables, whereas Mann–Whitney U test was employed to assess categorical variables. The AUC was calculated to compare the diagnostic performance of the models. The DeLong test was used to compare the differences between the models.

## 3 Results

### 3.1 Patient characteristics

This study enrolled a total of 213 patients with PTC, comprising 124 positive patients and 89 negative patients. There were no statistically significant differences between the training and validation groups in terms of age, gender, TSH, T3, T4, FT3, FT4, Tg, TgAb, TPOAb, size, margin, macrocalcification, microcalcification, orientation, C-TIRADS, multifocality and CLNM. The statistical summary of the basic clinical characteristics is presented in **Table 1**. The training and testing cohorts demonstrated comparable baseline characteristics.

**Table 1:** The clinical and descriptive semantic features of patients with PTC.

Parameters	Training cohort (N= 170)	Testing cohort (N= 43)	P-value
Age	44.50 ± 12.39	44.86 ± 13.48	0.203
Gender			0.772
Male	35 (20.6%)	8 (18.6%)	
Female	135 (79.4%)	35 (81.4%)	
TSH	2.03 ± 1.17	1.63 ± 0.92	0.226
T3	1.83 ± 0.38	1.88 ± 0.47	0.140
T4	107.62 ± 24.72	107.63 ± 19.33	0.923
FT3	4.96 ± 1.40	5.00 ± 1.19	0.986
FT4	16.67 ± 5.69	16.71 ± 2.51	0.373
Tg	30.68 ± 66.71	31.23 ± 45.56	0.701
TgAb	180.50 ± 616.70	99.71 ± 214.09	0.147
TPOAb	39.07 ± 92.29	35.83 ± 95.57	0.675
Size	1.08 ± 0.74	1.02 ± 0.59	0.461
Margin			0.901
Smooth	43 (25.3%)	11 (25.6%)	
Irregular	32 (18.8%)	9 (20.9%)	
Fuzzy	65 (38.2%)	14 (32.6%)	
ET	30 (17.6%)	9 (20.9%)	
Macrocalcification			0.605
Negative	110 (64.7%)	26 (60.5%)	
Positive	60 (35.3%)	17 (39.5%)	
Microcalcification			0.218
Negative	77 (45.3%)	15 (34.9%)	
Positive	93 (54.7%)	28 (65.1%)	
Orientation			0.241
Horizontal	74 (43.5%)	23 (53.5%)	
Longitudinal	96 (56.5%)	20 (46.5%)	
C-TIRADS			0.969
4A	11 (6.5%)	3 (7.0%)	
4B	59 (34.7%)	14 (32.6%)	
4C	54 (34.7%)	14 (32.6%)	
5	12 (7.1%)	2 (4.7%)	
6	34 (20.0%)	10 (23.3%)	
Multifocality			0.448
Negative	104 (61.2%)	29 (67.4%)	
Positive	66 (38.8%)	14 (32.6%)	

TSH, thyroid-stimulating hormone; T3, triiodothyronine; T4, thyroxine; FT3, free triiodothyronine; FT4, free thyroxine; Tg, thyroglobulin; TgAb, thyroglobulin antibody; TPOAb, thyroid peroxidase antibody; Size, the maximum diameter of the tumor as displayed on grayscale ultrasound images; C-TIRADS, Chinese thyroid imaging reporting and data system.

### 3.2 Radiomics model

The 27 unimodal ultrasound radiomics models constructed using nine algorithms based on three individual modalities are presented in **Table 2**. For the grayscale model (G model), elastography model (E model), and microflow model (M model), the optimal algorithms are MLP, LightGBM, and AdaBoost, respectively.

**Table 2:** Performance of the unimodal ultrasound radiomics models.

Model			AUC (95% CI)	ACC	SEN	SPE
Modality	Group	Algorithm				
Grayscale	Training Cohort	LR	0.794 (0.727 - 0.860)	0.735	0.742	0.726
		NaiveBayes	0.780 (0.711 - 0.849)	0.706	0.649	0.781
		SVM	0.710 (0.633 - 0.787)	0.671	0.577	0.795
		RandomForest	0.744 (0.674 - 0.815)	0.647	0.526	0.808
		ExtraTrees	0.763 (0.696 - 0.830)	0.694	0.794	0.562
		XGBoost	0.747 (0.682 - 0.812)	0.665	0.485	0.904
		LightGBM	0.776 (0.708 - 0.844)	0.688	0.608	0.795
		AdaBoost	0.794 (0.731 - 0.858)	0.647	0.402	0.973
		MLP *	0.813 (0.749 - 0.878)	0.741	0.660	0.849
	Testing Cohort	LR	0.782 (0.635 - 0.930)	0.698	0.667	0.750
		NaiveBayes	0.728 (0.562 - 0.894)	0.698	0.667	0.750
		SVM	0.671 (0.482 - 0.861)	0.721	0.741	0.687
		RandomForest	0.773 (0.639 - 0.907)	0.651	0.444	1.000
		ExtraTrees	0.623 (0.452 - 0.794)	0.535	0.333	0.875
		XGBoost	0.711 (0.569 - 0.853)	0.605	0.444	0.875
		LightGBM	0.773 (0.629 - 0.917)	0.651	0.593	0.750
		AdaBoost	0.615 (0.437 - 0.792)	0.512	0.556	0.437
		MLP *	0.799 (0.653 - 0.944)	0.767	0.741	0.812
Elastography	Training Cohort	LR	0.792 (0.725 - 0.859)	0.741	0.711	0.781
		NaiveBayes	0.733 (0.659 - 0.807)	0.653	0.495	0.863
		SVM	0.785 (0.717 - 0.853)	0.724	0.701	0.753
		RandomForest	0.769 (0.699 - 0.839)	0.700	0.701	0.699
		ExtraTrees	0.715 (0.640 - 0.789)	0.553	0.247	0.959
		XGBoost	0.743 (0.679 - 0.807)	0.671	0.515	0.877
		LightGBM *	0.800 (0.735 - 0.866)	0.712	0.598	0.863
		AdaBoost	0.768 (0.704 - 0.832)	0.618	0.392	0.918
		MLP	0.760 (0.690 - 0.831)	0.700	0.598	0.836
	Testing Cohort	LR	0.736 (0.564 - 0.908)	0.767	0.889	0.562
		NaiveBayes	0.758 (0.596 - 0.921)	0.698	0.704	0.687
		SVM	0.727 (0.556 - 0.898)	0.767	0.926	0.500
		RandomForest	0.765 (0.610 - 0.920)	0.651	0.630	0.687
		ExtraTrees	0.760 (0.615 - 0.906)	0.488	0.222	0.937
		XGBoost	0.579 (0.413 - 0.745)	0.488	0.407	0.625
		LightGBM *	0.775 (0.624 - 0.927)	0.698	0.704	0.687
		AdaBoost	0.653 (0.495 - 0.811)	0.395	0.037	1.000
		MLP	0.706 (0.528 - 0.884)	0.698	0.778	0.562
Microflow	Training Cohort	LR	0.751 (0.678 - 0.823)	0.682	0.639	0.740
		NaiveBayes	0.718 (0.641 - 0.795)	0.659	0.536	0.822
		SVM	0.742 (0.668 - 0.816)	0.676	0.629	0.740
		RandomForest	0.782 (0.714 - 0.851)	0.682	0.536	0.877
		ExtraTrees	0.705 (0.634 - 0.777)	0.576	0.289	0.959
		XGBoost	0.715 (0.645 - 0.785)	0.588	0.330	0.932
		LightGBM	0.757 (0.687 - 0.828)	0.659	0.485	0.890
		AdaBoost *	0.792 (0.728 - 0.857)	0.682	0.660	0.712
		MLP	0.742 (0.669 - 0.815)	0.688	0.732	0.630
	Testing Cohort	LR	0.655 (0.478 - 0.833)	0.674	0.630	0.750
		NaiveBayes	0.736 (0.555 - 0.918)	0.767	0.815	0.687
		SVM	0.660 (0.486 - 0.833)	0.651	0.630	0.687
		RandomForest	0.721 (0.548 - 0.894)	0.628	0.519	0.812
		ExtraTrees	0.616 (0.432 - 0.800)	0.558	0.519	0.625
		XGBoost	0.733 (0.567 - 0.899)	0.465	0.259	0.812
		LightGBM	0.641 (0.475 - 0.808)	0.558	0.444	0.750
		AdaBoost *	0.811 (0.666 - 0.956)	0.651	0.630	0.687
		MLP	0.650 (0.463 - 0.838)	0.698	0.815	0.500

AUC, area under the curve; ACC, accuracy; CI, confidence interval; SEN, sensitivity; SPE, specificity; LR, Logistic Regression; NaiveBayes, Naive Bayes Classifier; RandomForest, Random Forest Classifier; SVM, Support Vector Machine; ExtraTrees, Extremely Randomized Trees; XGBoost, Extreme Gradient Boosting; LightGBM, Light Gradient Boosting Machine; MLP, Multilayer Perceptron; AdaBoost, Adaptive Boosting; “ * ” denotes the algorithm that was ultimately selected.

Considering that the diagnostic approach in clinical practice relies primarily on grayscale ultrasound combined with other modalities, three combinations were applied to construct multimodal ultrasound radiomics models, including grayscale & elastography (G-E model), grayscale & microflow (G-M model), and grayscale & elastography & microflow (G-E-M model).

**Figure 4:**
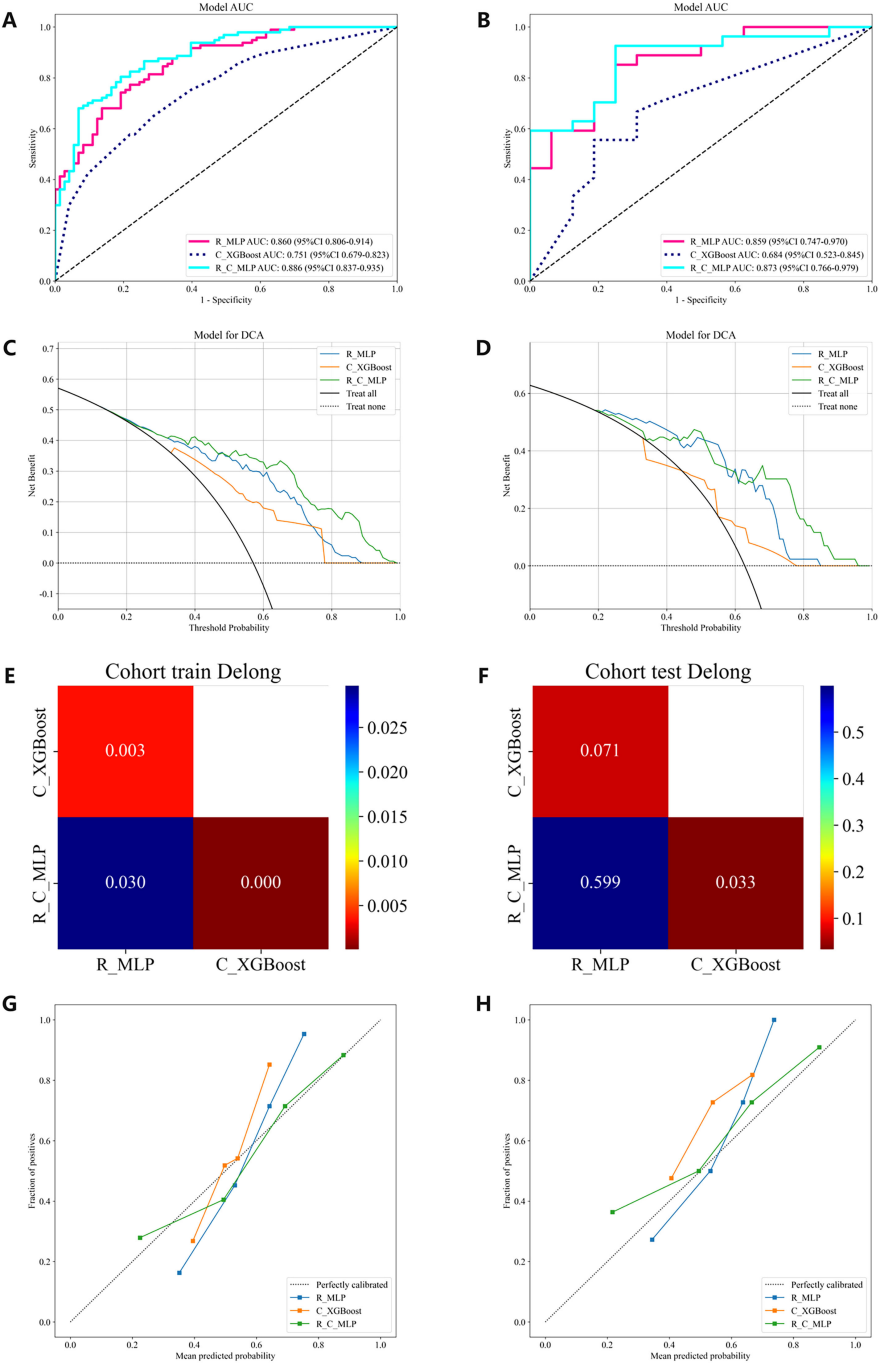
The ROC curves, DCA, Delong and Calibration curves for the R model, C model, and R-C model in both the training and testing cohorts. **(A)** ROC curves in the training cohort. **(B)** ROC curves in the testing cohort. **(C)** DCA in the training cohort. **(D)** DCA in the testing cohort. **(E)** The training cohort Delong. **(F)** The testing cohort Delong. **(G)** Calibration curves in the training cohort. **(H)** Calibration curves in the testing cohort. ROC, receiver operating characteristic; DCA, decision curve analysis.

The multimodal ultrasound models were constructed using the three algorithms above (MLP, LightGBM, and AdaBoost), and MLP achieved the highest AUC in all three multimodal ultrasound models. The G-E-MLP model achieved an AUC of 0.824 (95% CI, 0.764–0.884) in the training cohort and an AUC of 0.801 (95% CI, 0.655–0.947) in the testing cohort. The G-M-MLP model achieved an AUC of 0.833 (95% CI, 0.774–0.893) in the training cohort and an AUC of 0.815 (95% CI, 0.668–0.962) in the testing cohort. The G-E-M-MLP model was selected as the optimal R model, with an AUC of 0.860 (95% CI, 0.806–0.914) in the training cohort and an AUC of 0.859 (95% CI, 0.747–0.970) in the testing cohort. In the training set, the accuracy, sensitivity, and specificity were 0.771, 0.763, and 0.781, respectively, whereas in the testing set, the accuracy, sensitivity, and specificity were 0.791, 0.815, and 0.750, respectively, as shown in **Table 3**. The ROC curves and decision curves are shown in **Figure 4**.

**Table 3:** Performance of the multimodal ultrasound radiomics models.

Model			AUC (95% CI)	ACC	SEN	SPE
Modality	Group	Algorithm				
G-E	Training Cohort	LightGBM	0.769 (0.700 - 0.839)	0.665	0.588	0.767
		AdaBoost	0.799 (0.736 - 0.863)	0.682	0.515	0.904
		MLP *	0.824 (0.764 - 0.884)	0.747	0.701	0.808
	Testing Cohort	LightGBM	0.612 (0.437 - 0.788)	0.535	0.481	0.625
		AdaBoost	0.644 (0.473 - 0.814)	0.488	0.222	0.937
		MLP *	0.801 (0.655 - 0.947)	0.744	0.704	0.812
G-M	Training Cohort	LightGBM	0.809 (0.743 - 0.875)	0.747	0.763	0.726
		AdaBoost	0.801 (0.738 - 0.865)	0.724	0.804	0.616
		MLP *	0.833 (0.774 - 0.893)	0.771	0.845	0.671
	Testing Cohort	LightGBM	0.813 (0.676 - 0.949)	0.767	0.815	0.687
		AdaBoost	0.640 (0.461 - 0.819)	0.605	0.630	0.562
		MLP *	0.815 (0.668 - 0.962)	0.791	0.926	0.562
G-E-M ^#^	Training Cohort	LightGBM	0.764 (0.693 - 0.835)	0.653	0.557	0.781
		AdaBoost	0.821 (0.758 - 0.883)	0.729	0.711	0.753
		MLP *	0.860 (0.806 - 0.914)	0.771	0.763	0.781
	Testing Cohort	LightGBM	0.714 (0.556 - 0.872)	0.581	0.407	0.875
		AdaBoost	0.737 (0.570 - 0.904)	0.605	0.556	0.687
		MLP *	0.859 (0.747 - 0.970)	0.791	0.815	0.750

G-E, grayscale & elastography; G-M, grayscale & microflow; G-E-M, grayscale & elastography & microflow; AUC, area under the curve; ACC, accuracy; CI, confidence interval; SEN, sensitivity; SPE, specificity; LightGBM, Light Gradient Boosting Machine; AdaBoost, Adaptive Boosting; MLP, Multilayer Perceptron; “ ^#^ ” denotes the modality that was ultimately selected; “ * ” denotes the algorithm that was ultimately selected.

### 3.3 Clinical model

**Figure 5:**
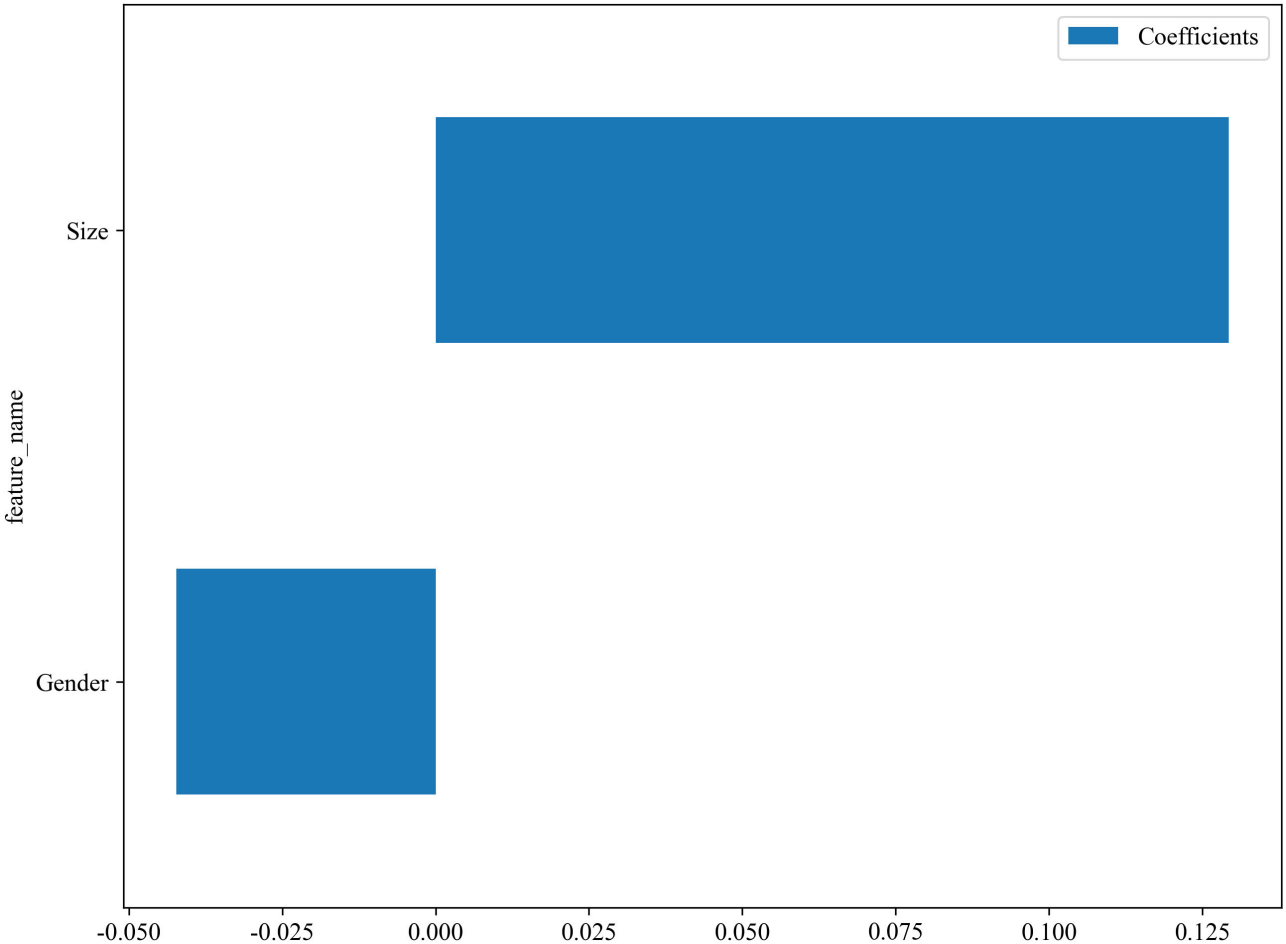
The factors included in the C models.

The C models were constructed using nine different algorithms. Among them, the clinical model built with the XGBoost algorithm demonstrated the best performance, achieving an AUC of 0.751 (95% CI, 0.679–0.823) in the training cohort and an AUC of 0.684 (95% CI, 0.523–0.845) in the testing cohort. In the training set, the accuracy, sensitivity, and specificity were 0.659, 0.577, and 0.767, respectively, while in the testing set, the accuracy, sensitivity, and specificity were 0.558, 0.407, and 0.812, respectively, as shown in **Table 4**. The factors ultimately included in the C model are shown in **Figure 5**, and the ROC curves and decision curves are shown in **Figure 4**.

**Table 4:** Performance of the optimal R model, the optimal C model, and the R-C models.

Model			AUC (95% CI)	ACC	SEN	SPE
Modality	Group	Algorithm				
R	Training Cohort	MLP	0.860 (0.806 - 0.914)	0.771	0.763	0.781
	Testing Cohort	MLP	0.859 (0.747 - 0.970)	0.791	0.815	0.750
C	Training Cohort	XGBoost	0.751 (0.679 - 0.823)	0.659	0.577	0.767
	Testing Cohort	XGBoost	0.684 (0.523 - 0.845)	0.558	0.407	0.812
R-C ^#^	Training Cohort	XGBoost	0.826 (0.765 - 0.887)	0.694	0.557	0.877
		MLP *	0.886 (0.837 - 0.935)	0.800	0.794	0.808
	Testing Cohort	XGBoost	0.654 (0.481 - 0.827)	0.581	0.556	0.625
		MLP *	0.873 (0.766 - 0.979)	0.837	0.889	0.750

R, radiomics; C, clinical; R-C, radiomics &clinical; AUC, area under the curve; ACC, accuracy; CI, confidence interval; SEN, sensitivity; SPE, specificity; XGBoost, Extreme Gradient Boosting; LightGBM, Light Gradient Boosting Machine; MLP, Multilayer Perceptron; “ ^#^ ” denotes the modality that was ultimately selected; “ * ” denotes the algorithm that was ultimately selected.

### 3.4 Clinical radiomics model

The R-C model was constructed using MLP, which is most suitable for the R model, and XGBoost, which is most suitable for the C model. The performances of the optimal R model, the optimal C model, and the R-C models are shown in **Table 4**. Ultimately, the R-C-MLP model demonstrated the best performance in both the training and testing cohorts, with AUCs of 0.886 (95% CI, 0.837–0.935) and 0.873 (95% CI, 0.766–0.979), respectively. In the training set, the accuracy, sensitivity, and specificity were 0.800, 0.794, and 0.808, respectively, while in the testing set, the accuracy, sensitivity, and specificity were 0.837, 0.889, and 0.750, respectively. **Figure 4** presents the ROC curves and DCA, illustrating the comparative analysis of the R model, C model, and R-C model as assessed by the Delong test. Additionally, the calibration curve in **Figure 4** illustrates the model’s goodness-of-fit.

**Figure 6:**
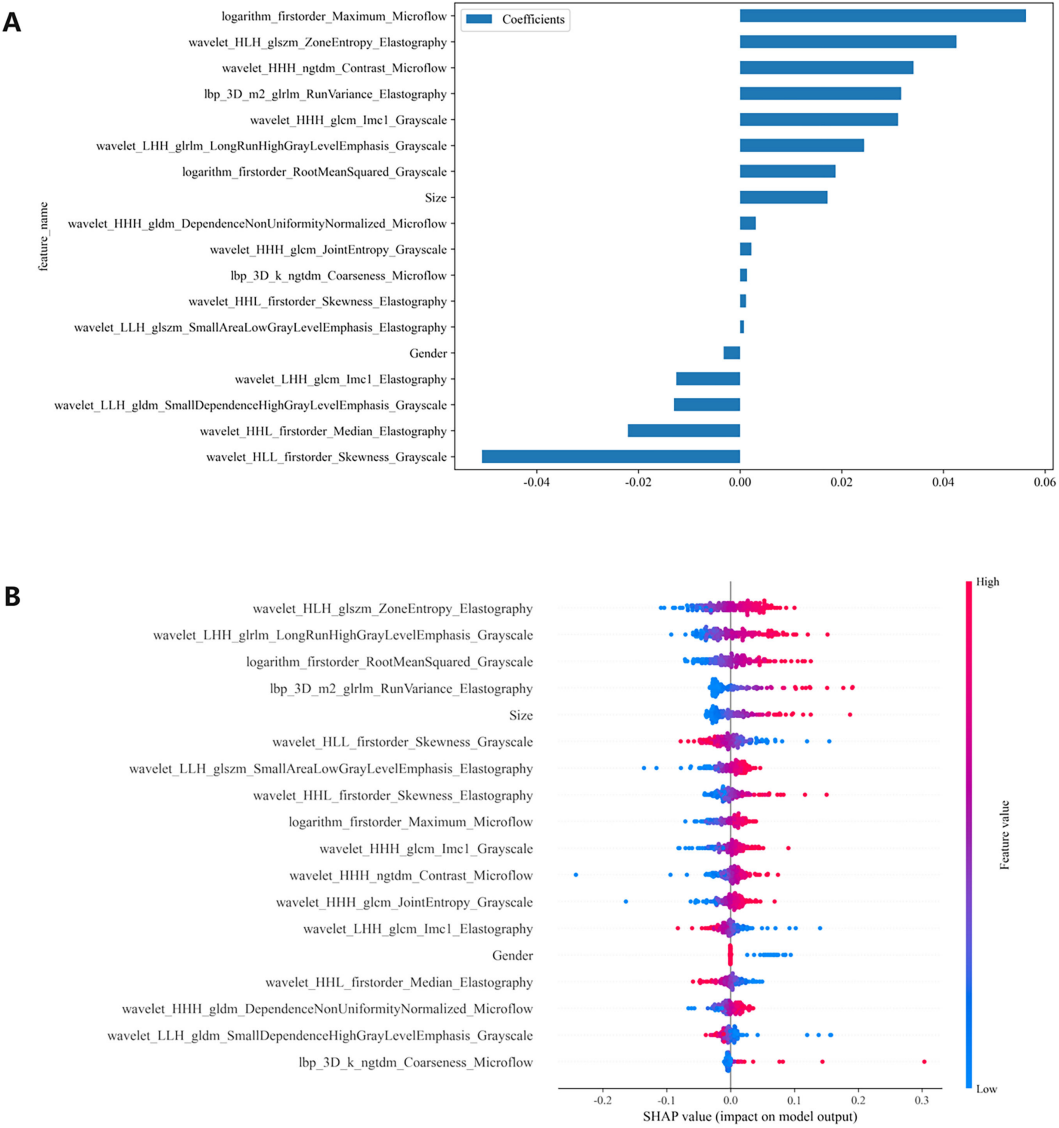
**(A)** The feature weight map presents all features and **(B)** the SHAP beeswarm plot visualizes feature impacts on prediction probability, where red and blue colors respectively indicate positive and negative directional influences. SHAP, Shapley Additive Explanations.

**Figure 7:**
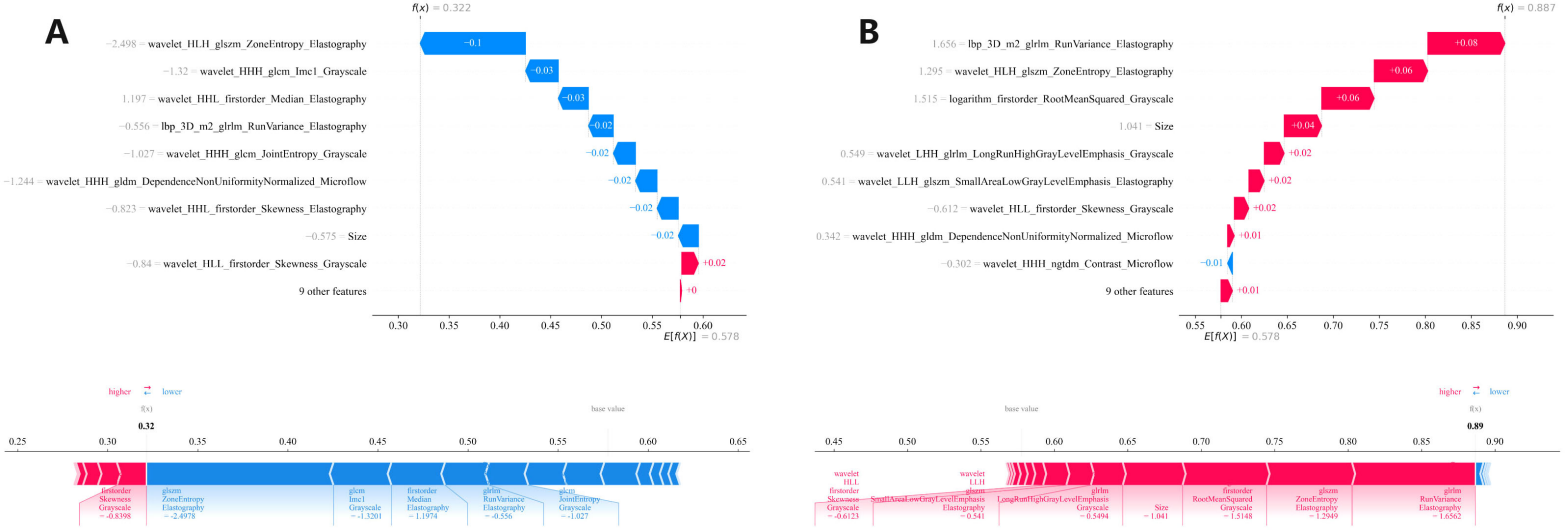
Two **(A, B)** local SHAP plots visually demonstrates the contribution of the features to the predicted probability for specific cases, with red and blue colors representing positive and negative influences, respectively. SHAP, Shapley Additive Explanations.

**Figure 8:**
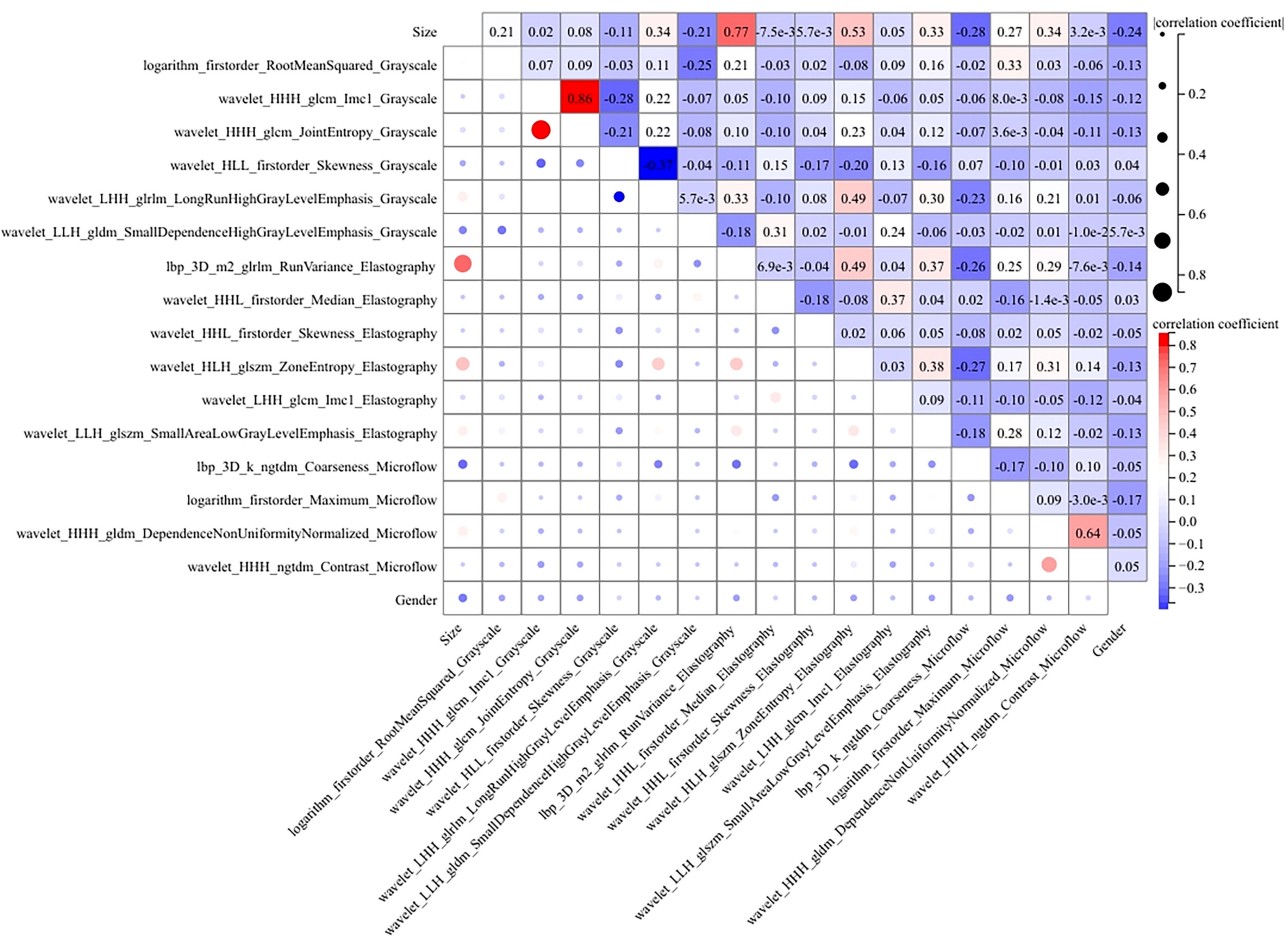
The heatmap of Pearson correlation for the features used in the R-C model. Lighter hues and smaller dot sizes indicate weaker feature correlations.

We conducted a comprehensive calculation of the overall and individual Shapley values for the R-C model to enhance its interpretability and facilitate its clinical application. For the overall visualization, **Figure 6** presents the feature weight map and SHAP Beeswarm plot. For individual visualization, **Figure 7** illustrates two typical cases, displaying the SHAP force plots. In addition, the Pearson correlations of the features included in the final model are shown in **Figure 8**.

## 4 Discussion

We developed and tested three types of models, including R models, C models and R-C models, for predicting the risk of developing CLNM in patients with PTC. The R models include unimodal, bimodal and trimodal ultrasound radiomics models, of which the trimodal ultrasound radiomics model trained using MLP yielded the best prediction. Compared with previous models, the optimal R model incorporates features from grayscale images, elastography images, and microflow images, while the R-C model further integrates clinical features, resulting in a greater AUC in both cohorts (25–28) Significantly, the novelty lies in the ability to assess the probability of CLNM risk preoperatively and noninvasively on the basis of comprehensive and detailed multimodal ultrasound image features. Compared with previous models, we included more ultrasound modalities and more precise ultrasound image features for prediction. The results fully demonstrate the significant clinical application value of ML models constructed by combining multimodal ultrasound radiomics features with clinical features in the preoperative assessment of CLNM in PTC patients. These models provide clinicians with more comprehensive and personalized imaging information, which is crucial for the selection of treatment strategies.

The R-C-MLP model demonstrated superior sensitivity in both the training (0.794) and validation (0.889) cohorts, significantly outperforming conventional ultrasound methods (sensitivity ≈0.55) for detecting CLNM. With accuracy exceeding 0.80 in both datasets, these results strongly support the clinical applicability of this model. After inputting each patient’s imaging data into the model, it generates an easy-to-interpret predicted probability of CLNM risk (as shown in **Figure 7**), which provides direct guidance for subsequent treatment planning. This model can serve as a diagnostic reference for radiologists, enabling either active monitoring or thermal ablation for patients predicted to have low CLNM risk. Furthermore, the model’s outcomes provide evidence-based guidance for clinicians’ therapeutic decision-making, facilitating personalized surgical approaches tailored to individual patients — a significant advancement over the current uniform pCLND protocol applied to all cases.

### 4.1 Prediction performance of clinical features

As the primary imaging method for determining CLNM, ultrasound typically uses the diameter of the lymph nodes and changes in internal echogenicity as criteria for abnormal detection (29). However, CLN images are susceptible to interference from neck anatomy. Consequently, the misdiagnosis rate is high for patients with LNM who do not exhibit obvious abnormalities, necessitating more sensitive predictive methods in clinical practice. In this study, the conventional ultrasound feature ultimately incorporated into the model was the maximum diameter of the tumor. **Figure 5** illustrates that a larger maximum tumor diameter is a contributing factor to CLNM, which aligns with findings from previous research (30, 31). In the vast majority of patients with PTC, the risk of LNM increases with increasing tumor size. Thus, tumor size indirectly reflects LNM. Gender is an important factor in the development of PTC. The percentage of women with PTC is significantly higher than that of men. While previous studies have shown that male gender is one of the factors contributing to the development of CLNM in patients with PTC (32, 33), which was also demonstrated in this study (**Figure 5**). The optimal C model established on the basis of tumor size and gender yielded AUC values of 0.751 and 0.684 for the training and testing cohorts, respectively (**Table 4**). However, the diagnostic performance is not sufficient to meet the requirements for clinical diagnostic applications.

### 4.2 Prediction performance of R models and R-C models

With the advent of radiomics, traditional imaging can be transformed into high-dimensional data for image analysis, thereby better quantifying lesion characteristics that are indistinguishable to the naked eye (34, 35) and reducing the subjectivity of diagnostic physicians (36, 37). Radiomics has been widely applied in various diseases, such as predicting tumor staging, tissue typing, and genetic status (38–40). This study incorporates microflow images, a novel technology not yet widely used in clinical practice, along with commonly used grayscale images and elastography images. The experimental results show that the R-MLP model performs well, with AUCs for the training and testing cohorts being 0.860 and 0.859, respectively, both exceeding those of the clinical model (**Figure 4**).

To further enhance diagnostic efficiency, a R-C-MLP model was developed by integrating clinical features with multimodal ultrasound radiomics features, achieving areas under the ROC curve of 0.886 and 0.873 for the training and testing cohorts, respectively. The inclusion of clinical features effectively improved the model’s accuracy, sensitivity, and specificity (**Table 4**), reflecting the role of clinical features in the noninvasive assessment of CLNM. The Delong test indicated that the differences between the R-C-MLP model and the C-MLP model in the training and testing cohorts were statistically significant, demonstrating that ultrasound radiomics can significantly contribute to the clinical diagnosis of CLNM (**Figure 4**). Moreover, the calibration curve presented in **Figure 4** further validates the predictive good fit of the R-C-MLP model.

### 4.3 Interpretation of the R-C MLP model

The MLP algorithm was eventually adopted in the construction of the fusion model. To explain the R-C MLP model, this study utilized SHAP to visualize the importance of model features and calculated SHAP values via game theory methods. SHAP values allocate the probability of model output to each feature, helping to understand the contribution of each feature to the prediction results, thereby making the model’s predictions more transparent and interpretable. The final model incorporated eighteen features, including 6 grayscale image features, 6 elastography image features, 4 microflow image features, and 2 clinical features. The weight of each feature in the model is shown in **Figure 6A**. The SHAP values of these features for each case are presented in **Figure 6B**. The visualization of Pearson correlation revealed that the correlations between grayscale features and elastography features, as well as between grayscale features and microflow features, were relatively low (below 0.5) (**Figure 8**). This finding indicates that elastography and microflow modalities can provide supplementary information to conventional grayscale ultrasound.

Furthermore, of the two bimodal models, the predictive performance of the G-M model was superior to that of the G-E model (**Table 3**), suggesting that although elastography is the more commonly used technique in clinical diagnostics today, microflow may have comparable or greater potential application. According to previous studies, the microflow patterns of thyroid nodules is associated with their malignancy (17). Moreover, several studies have demonstrated that the distribution and morphology of microvessels within tumor lesions are closely associated with tumor aggressiveness and microenvironment (41, 42). Ultrasound microflow imaging can visualize microvessels, providing a convenient method for assessing intratumoral microvasculature and offering new insights into tumor pathophysiology. In contrast, ultrasound elastography only reflects tissue stiffness changes and cannot provide additional information related to tumor progression. Therefore, models incorporating ultrasound microflow images may yield superior predictive performance.

In the R-C MLP model, the microflow feature logarithm_firstorder_Maximum_Microflow had the highest weight, indicating its significant contribution to the model’s outcomes (**Figure 6A**). Additionally, the microflow feature wavelet_HHH_ngtdm_Contrast_Microflow also exhibited a relatively high weight. The feature logarithm_firstorder_Maximum_Microflow quantifies the gray-level distribution characteristics of an image. In microflow images, a higher value of this feature indicates richer microvascular distribution within the lesion. The feature wavelet_HHH_ngtdm_Contrast_Microflow enhances fine structural details and measures local contrast in the image. For microflow images, an elevated value of this feature may suggest more complex microvascular morphology in the lesion. Our study revealed that higher values of these two features correlate with an increased risk of CLNM, implying that lesions with more abundant microflow and more complex microflow morphology are more likely to exhibit metastatic spread — a finding consistent with previous research (17). The results demonstrated that microflow ultrasound can provide a completely new perspective to complement conventional ultrasound in the preoperative diagnosis of CLNM. Moreover, the feature wavelet_HLH_glszm_ZoneEntropy_Elastography from elastography images also contributed significantly to the model performance. Higher values of this feature indicate greater heterogeneity in tissue elasticity distribution, reflecting calcification patterns and stiffness variations. Our findings demonstrated that intratumoral elasticity heterogeneity is significantly associated with an increased risk of CLNM, which aligns consistently with prior published studies (9).

### 4.4 Limitations and research prospects

This study has several limitations. It should be noted that the retrospective, single-center design of this study lead to a potential selection bias that may influence our results. Since microflow imaging technology has not yet been widely applied in clinical settings, obtaining standardized images of all three modalities simultaneously is challenging in practice. Therefore, our study was limited by a relatively small sample size, and conducting multi-center research currently presents significant practical challenges. Although internal validation showed that the model has stable diagnostic performance, future multi-center prospective studies are needed to further validate the model’s generalizability, particularly its reproducibility across different regions, devices, and operators. We hope that with the increasing clinical adoption of microflow ultrasound imaging, multicenter studies with larger sample sizes can be conducted in the future.

In addition, although this study has used SHAP to perform a visual analysis of the model, users still need to undergo training in data interpretation before implementing the proposed model in clinical practice, so that clinicians can better accept the prediction results. Moreover, the clinical relevance of the selected features and their biological significance in relation to CLNM development could not be thoroughly investigated within the scope of the current study. For future research, we intend to increase the sample size to enable a more in-depth investigation of the correlation between radiomics features and cellular pathology.

With the rapid advancement of artificial intelligence technologies, an increasing number of novel models are being applied to medical image analysis (43–45). Moving forward, we plan to explore additional methodologies to further refine and enhance the interpretability of our current model.

## 5 Conclusion

In conclusion, this study proposes a fusion model based on clinical and multimodal ultrasound radiomics features, which has high accuracy in predicting CLNM in PTC patients. This model included grayscale ultrasound, elastography ultrasound and microflow ultrasound. Our findings confirm that microflow images can be used as a basis for preoperative assessment of CLNM, and may be included in the diagnostic criteria along with conventional ultrasound in the future. This model will provide clinicians with more comprehensive and personalized imaging information, enabling noninvasive assessment of CLNM status, which is highly important for the selection of treatment strategies.

## Data Availability

The raw data supporting the conclusions of this article will be made available by the authors, without undue reservation.
